# Butylidenephthalide Abrogates the Snail-Induced Cancer Stemness in Oral Carcinomas

**DOI:** 10.3390/ijms23116157

**Published:** 2022-05-31

**Authors:** Pei-Yin Chen, Shih-Chi Chao, Pei-Ling Hsieh, Yi-Wen Liao, Pei-Ming Chu, Horng-Jyh Harn, Cheng-Chia Yu

**Affiliations:** 1 School of Dentistry, Chung Shan Medical University, Taichung 402, Taiwan; send8034@hotmail.com; 2 Department of Dentistry, Chung Shan Medical University Hospital, Taichung 402, Taiwan; 3 Institute of Oral Sciences, Chung Shan Medical University, Taichung 402, Taiwan; h5l4g4fu6123@gmail.com; 4 Department of Medical Research and Education, Lo-Hsu Medical Foundation, Lotung Poh-Ai Hospital, Yilan 265, Taiwan; 5 Department of Anatomy, School of Medicine, China Medical University, Taichung 404, Taiwan; plhsieh@mail.cmu.edu.tw (P.-L.H.); pmchu@mail.cmu.edu.tw (P.-M.C.); 6 Department of Medical Research, Chung Shan Medical University Hospital, Taichung 402, Taiwan; rabbity0225@gmail.com; 7 Buddhist Tzu Chi Bioinnovation Center, Tzu Chi Foundation, Hualien 970, Taiwan; 8 Bioinnovation Center, Buddhist Tzu Chi Medical Foundation, Hualien 970, Taiwan; 9 Department of Pathology, Buddhist Tzu Chi General Hospital and Tzu Chi University, Hualien 970, Taiwan

**Keywords:** butylidenephthalide, oral cancer, cancer stem cells, ALDH1, CD44, Snail

## Abstract

Oral cancer is one of the most common cancers worldwide, especially in South Central Asia. It has been suggested that cancer stem cells (CSC) play crucial roles in tumor relapse and metastasis, and approaches to target CSC may lead to promising results. Here, aldehyde dehydrogenase 1 (ALDH1) and CD44 were utilized to isolate CSCs of oral cancer. Butylidenephthalide, a bioactive phthalide compound from *Angelica sinensis*, was tested for its anti-CSC effects. MTT assay showed that a lower concentration of butylidenephthalide was sufficient to inhibit the proliferation of patient-derived ALDH1^+^/CD44^+^ cells without affecting normal cells. Administration of butylidenephthalide not only reduced ALDH1 activity and CD44 expression, it also suppressed the migration, invasion, and colony formation abilities of ALDH1^+^/CD44^+^ cells using a transwell system and clonogenic assay. A patient-derived xenograft mouse model supported our in vitro findings that butylidenephthalide possessed the capacity to retard tumor development. We found that butylidenephthalide dose-dependently downregulated the gene and protein expression of Sox2 and Snail. Our results demonstrated that overexpression of Snail in ALDH1^-^/CD44^-^ (non-CSCs) cells induced the CSC phenotypes, whereas butylidenephthalide treatment successfully diminished the enhanced self-renewal and propagating properties. In summary, this study showed that butylidenephthalide may serve as an adjunctive for oral cancer therapy.

## 1. Introduction

Oral cancer involves malignancies of the oral cavity and lip region, which belong to head and neck cancers, with approximately 90% being identified as squamous cell carcinomas (OSCC). In the malignant tumor progression, OSCC mostly originates from the antecedent dysplastic oral epithelial lesions, such as leukoplakia and erythroplakia, due to their elevated risk of developing an invasive carcinoma [1]. According to the GLOBOCAN 2018 estimation, oral cancer is highly frequent in Southern Asia and is the leading cause of cancer death among men in India and Sri Lanka [2]. It has been shown that the rate of nodal relapses was around 78% in patients who had the therapeutic surgery, and the rate of non-nodal recurrences (local or distant metastasis or second primary tumors) was about 52% for oral cancer patients who received elective surgery [3]. Another study also revealed that a significant number of patients was diagnosed with distant metastases, which often led to dismal prognoses as the median time to death was ±4 months [4]. Hence, it is imperative to develop an effective treatment to minimize the size of tumor or delay the spread of disease.

Metastases occur when tumor cells are disseminated from the primary tumor and subsequently spawn new colonies It is known that cells that underwent an epithelial-to-mesenchymal transition (EMT) acquired invasive properties and stem cell-like features, which contributed to the multi-step process of the invasion–metastasis cascade. These cells were regarded as cancer stem cells (CSCs) since they were able to self-renew and generate new tumors [5]. Emerging evidence indicates that CSCs cause cancer recurrence and metastases (see review [6]). Therefore, it has been considered that the combination of conventional treatments with CSC-targeting agents may be an effective strategy for the eradication of cancer. To date, several methods have been used to identify CSCs in oral cancer, such as the positivity of CD44 [7], CD133 [8], and aldehyde dehydrogenase (ALDH) activity [9]. These approaches also have been applied to examine the anti-CSC effects of various adjunct therapies, such as photodynamic therapy [10] or natural products [11].

A traditional Chinese medicine, *Angelica sinensis* (also called Dang Gui), possesses numerous pharmacological and therapeutic effects, including improved cardiovascular function [12], protection from pancreatic islets failure [13], enhanced immune responses to infection [14], and suppressing malignant tumor progression [15]. Butylidenephthalide, among the major compounds derived from *Angelica sinensis*, has been reported to exert an anti-angiogenic activity by suppressing the WD repeat and SOCS box-containing protein 1 (WSB-1)/von Hippel–Lindau (pVHL)/hypoxia-inducible factor-1α (HIF-1α)/vascular endothelial growth factor (VEGF) signaling axis in bladder cancer in vitro and in vivo [16]. Another study indicated that exposure to butylidenephthalide positively correlated with a lower incidence of bladder cancer in Taiwanese patients [17]. The administration of butylidenephthalide induced apoptosis in human bladder cancer cells, leading to the inhibition of cell migration and invasion and drug resistance. Treatment with butylidenephthalide at 100 and 200 mg/kg doses significantly suppressed bladder tumor growth in xenograft animal models. At the same dose, butylidenephthalide also diminished the growth rate and induced the intrinsic apoptosis of serous ovarian CSCs’ tumor in vivo [18]. Butylidenephthalide has been reported to be allowed to cross the blood–brain barrier and, thus, inhibited glioblastoma progression via diverse mechanisms, including the inhibition of tumor growth and induction of tumor apoptosis and senescence [19]. Encapsulation by lipopolyplexes maintained the activity and cellular uptake of butylidenephthalide that enhanced the cell apoptosis, cell cycle arrest, and drug sensitivity, thereby increasing the cytotoxicity of colorectal cancer cells [20]. In brain tumor, butylidenephthalide could trigger both p53-dependent and -independent pathways for apoptosis of glioblastoma multiforme cells [21]. Another study demonstrated that butylidenephthalide inhibited the stemness features and EMT-related molecules in glioblastoma CSCs through the suppression of the AXL/enhancer of the zeste homolog 2 (EZH2) pathway [22]. Additionally, butylidenephthalide has been shown to downregulate matrix metalloproteinase activity, which is associated with cancer cell invasion [23]. In the premalignant condition of the oral cavity, the administration of butylidenephthalide also suppressed numerous EMT-associated markers and impeded the progression of fibrogenesis [24]. Nevertheless, the effect of butylidenephthalide on the CSCs of oral cancer has not been investigated.

In the current study, we aimed to examine whether butylidenephthalide exhibited anti-CSC properties using patient-derived ALDH1^+^/CD44^+^ oral cancer cells. We sought to assess several cancer stemness features in vitro and the tumorigenic potential in vivo after butylidenephthalide treatment. Subsequently, we revealed that a zinc-finger transcription factor, Snail, was implicated in the anti-CSCs’ effect of butylidenephthalide.

## 2. Results

### 2.1. The Cytotoxicity of Butylidenephthalide on Normal Human Oral Keratinocyte (NHOK) and Patient-Derived ALDH1^+^/CD44^+^ Cells

**Figure 1:**
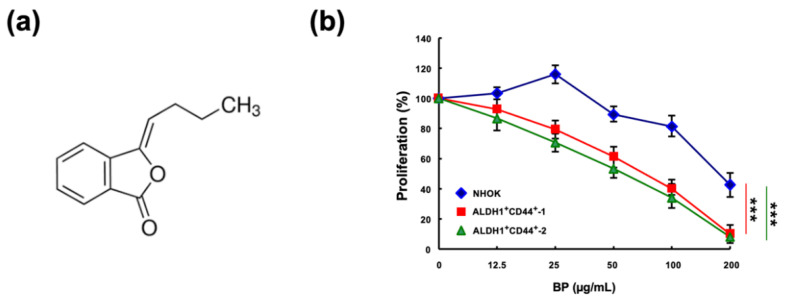
The cytotoxic effect of butylidenephthalide on the viability of normal human oral keratinocyte (NHOK) and patient-derived ALDH1^+^/CD44^+^ cells. (**a**) Chemical structures of butylidenephthalide; MTT assay was utilized to examine the cell viability of (**b**) NHOK and two patient-derived ALDH1^+^/CD44^+^ cells in response to butylidenephthalide at 12.5, 25, 50, 100, and 200 μg/mL. Results are presented as means ± SD of three independent experiments; *** p < 0.001 compared to NHOK. Butylidenephthalide (BP).

To investigate the effect of butylidenephthalide on the cell viability of the NHOK and patient-derived ALDH1^+^/CD44^+^ cells, cell proliferation was examined after a 48 h treatment of butylidenephthalide using a 3-(4,5-Dimethylthiazol-2-yl)-2,5-diphenyltetrazolium bromide (MTT) assay, and 50% inhibitory concentration (IC_50_) values were calculated to quantitatively evaluate the drug responses. IC_50_ values of the NHOK and patient-derived ALDH1^+^/CD44^+^ cells-1 and cells-2 were 176.8 ± 20.6, 56.4 ± 4.3, and 64.5 ± 6.4 μg/mL, respectively (Figure 1). This result showed that a lower concentration of butylidenephthalide was sufficient to exert a cytotoxic effect and reduce the cell viability of ALDH1^+^/CD44^+^ cells without damaging the NHOK. The butylidenephthalide used in the following experiments was in this concentration range to examine if it possessed an anti-CSCs’ property.

### 2.2. Butylidenephthalide Downregulates the Percentage of Cancer Stem Cells in Patient-Derived ALDH1^+^/CD44^+^ Cells

**Figure 2:**
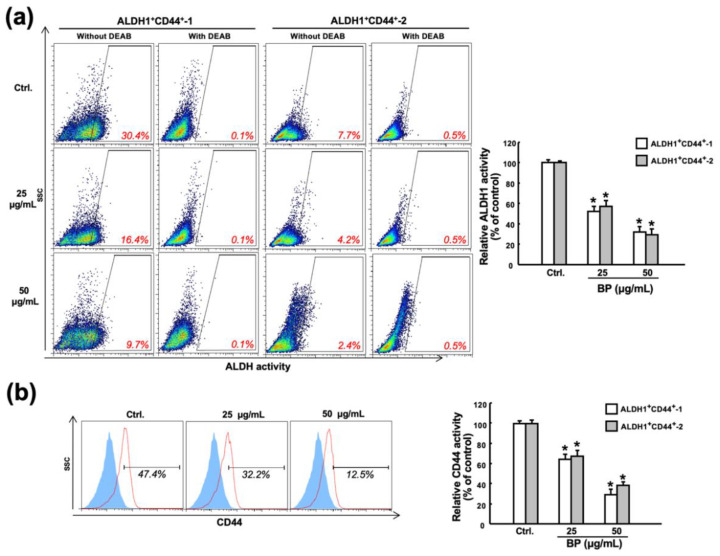
Butylidenephthalide suppresses the expression of CSC markers in two patient-derived ALDH1^+^/CD44^+^ cells. (**a**) Flow cytometry analyses of two patient-derived ALDH1^+^/CD44^+^ cells treated with various concentrations of butylidenephthalide for 48 h followed by incubation with ALDEFLUOR kit (to measure an ALDH1 enzymatic activity) in the presence of 4-diethylaminobenzaldehyde (DEAB, a specific ALDH1 inhibitor) as a control or (**b**) anti-CD44 antibody. Results are presented as means ± SD of three independent experiments; * *p* < 0.05 compared to Ctrl (control group containing 0.1% DMSO without butylidenephthalide). Butylidenephthalide (BP).

In order to investigate the effect of butylidenephthalide on the expression of cancer stemness markers, we tested the proportion of cells that express ALDH1 or CD44 after treatment with various concentrations of butylidenephthalide. ALDH1 activity has been proven to be indicative of radioresistance in CSCs of prostate cancer as it promoted an enhanced DNA repair capacity and gain of the epithelial-to-mesenchymal transition (EMT) features [25]. We found that the number of cells expressing ALDH1 dropped significantly in response to 25 and 50 μg/mL of butylidenephthalide (Figure 2a). As for CD44, it was revealed to maintain the self-renewal capacity of CSCs through the inhibition of phosphorylated glycogen synthase kinase 3β (pGSK3β) in head and neck cancers [26]. We showed that butylidenephthalide dose-dependently decreased the percentage of CD44^+^ cells (Figure 2b). These findings indicated that butylidenephthalide possesses the capacity to reduce the existence of CSCs and may mitigate the phenotypes of CSCs.

### 2.3. Butylidenephthalide Diminishes Various Stemness Phenotypes in Patient-Derived ALDH1^+^/CD44^+^ Cells

**Figure 3:**
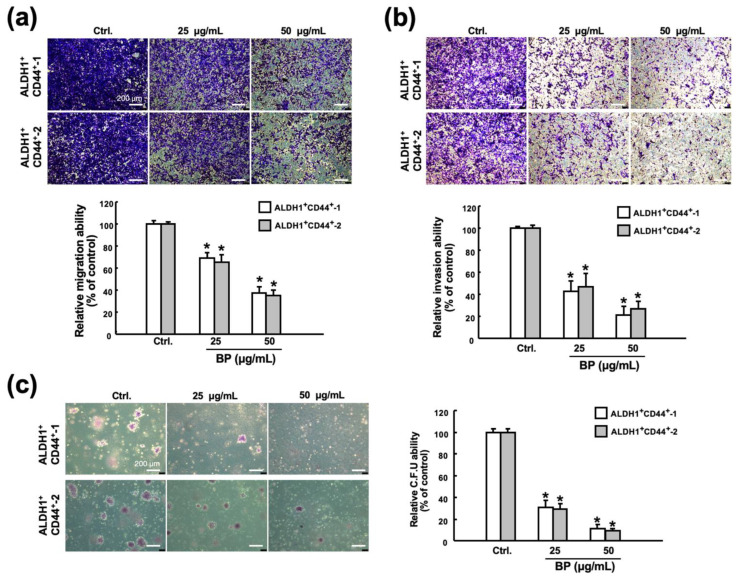
Butylidenephthalide eliminates the phenotypes of CSCs. The transwell culture system was employed to assess the (**a**) migration and (**b**) invasion capacities of two patient-derived ALDH1^+^/CD44^+^ cells in response to butylidenephthalide treatment. (**c**) Colony forming ability of ALDH1^+^/CD44^+^ cells was examined by clonogenic assay. Results are presented as means ± SD of three independent experiments; * *p* < 0.05 compared to Ctrl (control group containing 0.1% DMSO without butylidenephthalide). Butylidenephthalide (BP). Scale bar indicate 200 μm.

In an attempt to assess the impact of butylidenephthalide on the features of ALDH1^+^/CD44^+^ cells, we examined their migration, invasion, and colony forming capacities since ALDH1^+^ [27] or CD44^+^ [28] head and neck cancer cells exhibited higher cancer stemness properties. Moreover, it was demonstrated that ALDH1 correlated with the metastatic capacity, and CD44 was associated with tumorigenesis [29]. Our findings showed that butylidenephthalide downregulated their migration ability (Figure 3a), and the proportion of invading cells were also decreased in the butylidenephthalide-treated cells compared to the no-treatment group (Figure 3b). Moreover, a clonogenic assay revealed that the ability of ALDH1^+^/CD44^+^ cells to expand was dramatically suppressed (Figure 3c). These results demonstrated that the administration of butylidenephthalide diminished the propagating properties in a dose-dependent manner in vitro.

### 2.4. Administration of Butylidenephthalide Retards Tumor Growth in Patient-Derived Xenograft Model

**Figure 4:**
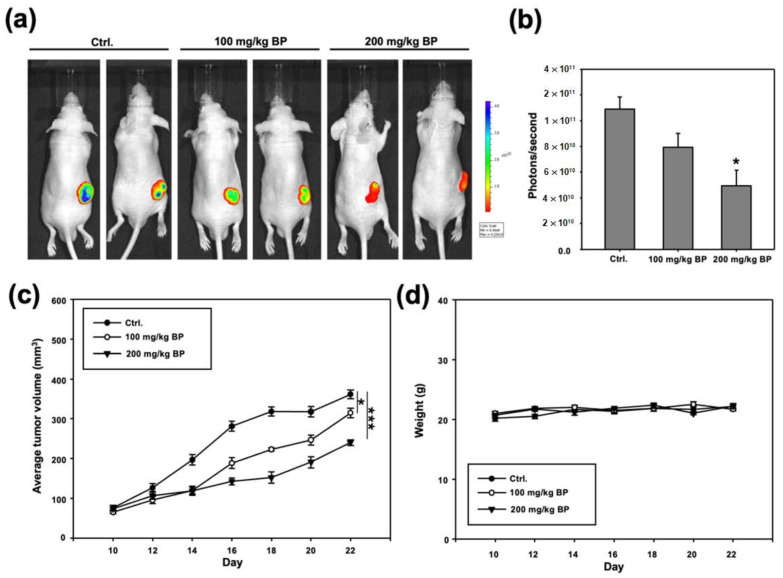
Butylidenephthalide inhibits in vivo tumorigenicity in the ALDH1^+^/CD44^+^-1-transplanted immunocompromised mice. After implantation of ALDH1^+^/CD44^+^ cells subcutaneously, BALB/c nude mice receiving various concentrations of butylidenephthalide were analyzed for the bioluminescence signal using IVIS imaging system. (**a**) The representative image and (**b**) quantitative analysis of signal intensity at day 22 are shown; (**c**) tumor volume; (**d**) body weight. The emitted signal by the implanted cells was monitored for 22 days (each group, *n* = 5). Results are means ± SD; * *p* < 0.05 compared to Ctrl (control group; administration with normal saline without butylidenephthalide). Butylidenephthalide (BP).

To obtain the in vivo evidence for the therapeutic effect of butylidenephthalide, the patient-derived xenograft (PDX) model was established and two different dose groups (100 and 200 mg/kg daily, given as a single dose, by subcutaneous injections of a vehicle alone or the indicated amount of butylidenephthalide) were tested. Results from bioluminescence imaging (BLI) demonstrated that the bioluminescence intensity of photons emitted from the tumors was lower in the butylidenephthalide-treated mice (Figure 4a,b). Moreover, a higher dose of butylidenephthalide exhibited a better potential to retard the growth of tumors (Figure 4c) while not affecting the mice’s body weight (Figure 4d). Altogether, these findings suggest that butylidenephthalide possesses the anti-CSC effects in vitro and in vivo.

### 2.5. Butylidenephthalide Reduces the Expression of Sox2 and Snail in Patient-Derived ALDH1^+^/CD44^+^ Cells

**Figure 5:**
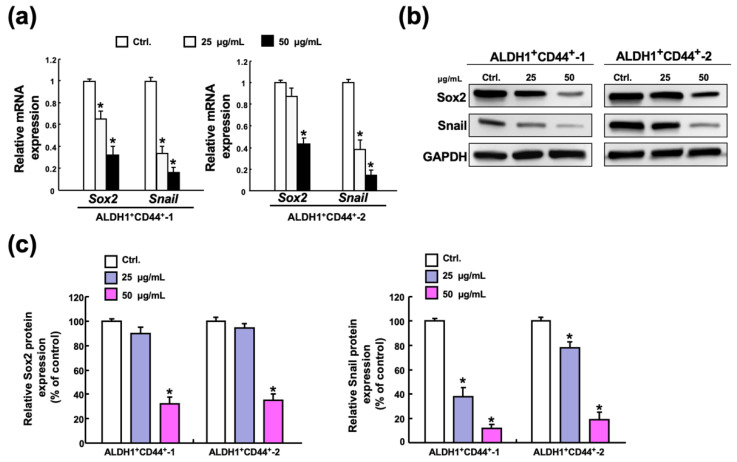
Administration of butylidenephthalide represses the expression of Sox2 and Snail. (**a**) Gene and (**b**) protein expression levels of stemness marker Sox2 and EMT marker Snail were both downregulated in the ALDH1^+^/CD44^+^ cells following treatment at various concentrations of butylidenephthalide. (**c**) The relative level of indicated protein expression was measured by densitometer and normalized against GAPDH. The control was set as 100%. Optical density values represent the mean ± SD of three independent experiments; * *p* < 0.05 compared to Ctrl (control group containing 0.1% DMSO without butylidenephthalide).

To explore the possible mechanism underlying the anti-CSC property of butylidenephthalide, the expression levels of the stemness marker Sox2 (sex-determining region Y (SRY)-Box2) and the EMT marker Snail were measured as these two factors have been shown to regulate several CSC features, such as self-renewal and tumorigenicity, in head and neck cancers [30,31,32]. The relative mRNA expression levels of Sox2 and Snail were dose-dependently reduced in both ALDH1^+^/CD44^+^ cells (Figure 5a). Likewise, the inhibited protein expression of Sox2 and Snail was observed, and it appeared that the expression of Snail was downregulated by butylidenephthalide to a greater degree than was Sox2 (Figure 5b,c). Hence, we extrapolated that butylidenephthalide may exert its inhibitory effects through the suppression of Snail.

### 2.6. Butylidenephthalide Mitiagtes the Stemness Phenotypes via Suppression of Snail in Oral Cancer Stem Cells

**Figure 6:**
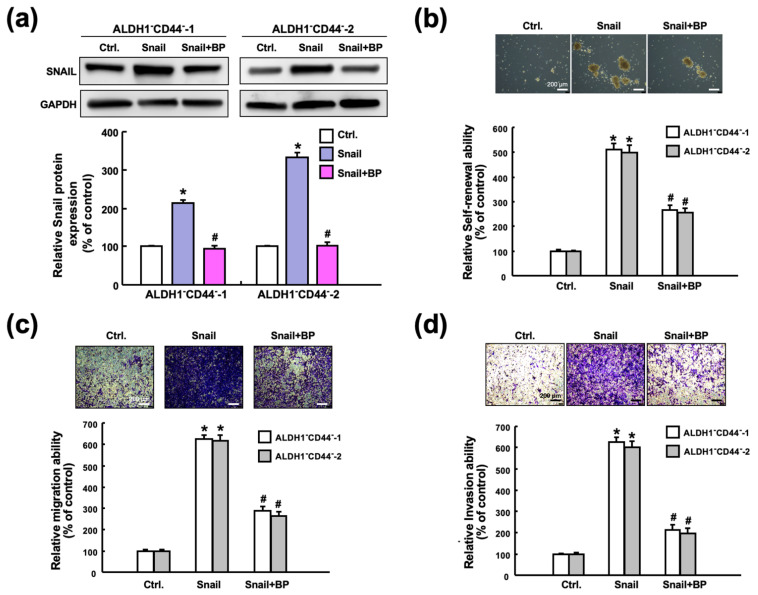
Butylidenephthalide diminishes numerous characteristics of CSCs through downregulation of Snail. (**a**) Overexpression of Snail in ALDH1^−^/CD44^−^ cells followed by treatment with or without butylidenephthalide. The enhanced (**b**) self-renewal, (**c**) migration, and (**d**) invasion abilities in the Snail-overexpressing ALDH1^−^/CD44^−^ cells were all inhibited after butylidenephthalide treatment. Data are shown as the mean ± SD of three independent experiments; * *p* < 0.05 compared to Ctrl (control group; transfection with control vector); # *p* < 0.05 compared to the Snail-overexpressing ALDH1^−^/CD44^−^ cells without-treatment group. Butylidenephthalide (BP). Scale bar indicate 200 μm.

To verify our hypothesis, we overexpressed Snail in ALDH1^−^/CD44^−^ cells (non-CSCs) (Figure 6a) and showed that upregulation of Snail in non-CSCs induced CSC properties, including higher self-renewal (Figure 6b), migration (Figure 6c), and invasion capacities (Figure 6d). Next, we demonstrated that butylidenephthalide attenuated the enhanced self-renewal and propagating abilities of Snail-overexpressing cells via the suppression of Snail (Figure 6b–d). Collectively, these results showed that butylidenephthalide was able to hinder the malignant progression through the downregulation of Snail.

## 3. Discussion

Natural compounds with potential therapeutic usage have been widely investigated over the past few decades. Several studies have shown that butylidenephthalide holds a potential for attenuating tumor progression. For instance, it has been shown that butylidenephthalide caused mitochondria-mediated apoptosis of gastric cancer cells with the upregulated expression regulated in development and DNA damage responses 1 (REDD1) expression and mTOR inhibition [33]. As for oral cancer, a derivative of n-butylidenephthalide, (Z)-N-(2-(dimethylamino)ethyl)-2-(3-((3-oxoisobenzofuran-1(3H)-ylidene)methyl)phenoxy)acetamide (PCH4), has been found to induce oral cancer cell apoptosis via the enhancement of nuclear receptor 4A1 (Nur77) translocation from the nucleus to the cytoplasm [34]. Nevertheless, the effects of butylidenephthalide on CSCs still remain to be elucidated. In the present study, we examined its impact on the cancer stemness traits of oral cancer CSCs in vitro and in vivo, and showed that butylidenephthalide inhibited the expression of Sox2 and Snail in the ALDH1^+^/CD44^+^ cells. This result is consistent with a previous report by Yen et al. showing that butylidenephthalide was able to downregulate Sox2 and octamer-binding transcription factor 4 (Oct4) in the CD133+ CSCs of glioblastoma [22].

Sox2 has been known as a crucial factor that participates in embryonic development and CSC maintenance [35]. In pancreatic cancer cells, overexpression of Sox2 not only increases the expression of ALDH1 and CD44 but also reduces the expression of epithelial marker E-cadherin through directly binding to the promoter of Snail [36]. Another study showed that Snail silencing decreased the sphere and colony forming capacity as well as the expression of ALDH and Sox2 [37]. In head and neck cancers, Lee et al. demonstrated that ablation of Sox2 in CSCs attenuated various stemness traits, including self-renewal and in vivo tumorigenicity. They showed that knockdown of Sox2 led to lower invasion capacity via the downregulation of Snail (but not Twist or Slug) [38]. Results from another group suggested that the silencing of Sox2 suppressed drug-resistance and anti-apoptotic genes, and they observed that the expression level of Snail, Slug, Twist, and vimentin were repressed in the CSCs of oral cancer [39]. On the other hand, it was demonstrated that the gene expression of Sox2 was upregulated in the Snail-overexpressing oral cancer cells, but the changes at the protein level were not significant (only Nanog, Bmi1, and ABCG2 were increased) [40]. It appeared that Sox2 was able to promote EMT through the direct activation of Snail, but the modulation of Snail may not result in dramatic alteration of Sox2 even though Snail was critical to the maintenance of Sox2. Our previous work demonstrated that butylidenephthalide suppressed the expression of various EMT markers and impeded the binding of Snail to the promoter of α-smooth muscle actin [24]. Therefore, it seems reasonable to observe that Snail was inhibited by butylidenephthalide to a greater degree than Sox2.

Accumulating studies have revealed that Snail was implicated in cancer stemness. It was previously reported that ALDH1^+^ cells endogenously co-expressed Snail, and the silence of Snail significantly decreased the expression of ALDH1, diminished CSC properties, and blocked the tumorigenic abilities of CD44^+^/ALDH1^+^ cells [30]. Snail also was found to increase CD44s in ovarian cancer cells through directly binding to E-boxes in the ESRP1 (epithelial splicing regulatory protein 1) promoter [41]. CD44 has been shown to be required for self-renewal of CSCs and it could regulate the phosphorylation of glycogen synthase kinase 3β (pGSK3β), which affects the expression of several stem cell markers, such as Sox2 [26]. Additionally, the depletion of CD44 has been demonstrated to effectively prevent circulating tumor cell aggregation, which downregulates the tumorigenesis and metastasis of breast cancer [42]. Interestingly, a recent report showed that the downregulation of CD44 abrogated the Snail expression of ovarian cancer cells [43]. These results suggest that the regulation of Snail may lead to changes in ALDH1 or CD44 expression. In line with these findings, we showed a concomitant reduction in Snail and ALDH1/CD44 in CSCs after butylidenephthalide treatment. Moreover, we demonstrated that the ectopic expression of Snail induced the self-renewal, migration, and invasion abilities in non-CSCs. This result was consistent with a previous study showing the introduction of Snail elicited a CSC-like phenotypic change and enhanced cell migration and invasion of head and neck cancers [44]. With the treatment of butylidenephthalide, the higher self-renewal ability and aggressiveness induced by Snail were markedly downregulated. In addition, we showed the inhibitory effect of butylidenephthalide on in vivo tumor growth, which was in agreement with several studies [33,45].

**Figure 7:**
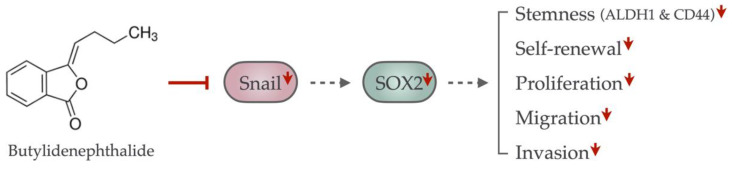
The graphic abstract of the present study illustrates the mechanism of butylidenephthalide to eliminate oral CSCs by suppressing the Snail-induced cancer stemness properties. The administration of butylidenephthalide suppressed the Snail expression, resulting in the downregulation of SOX2 expression, thereby abolishing the stemness markers’ expression, self-renewal, proliferation, migration, and invasion of oral CSCs. Red, solid line with vertical head indicates the inhibiting effect; gray, dotted line with arrowhead indicates the weakened increasing effect; red arrowhead indicates the downregulation.

In conclusion, we demonstrated that butylidenephthalide may serve as a suitable adjunct for oral cancer treatment as it was able to eliminate CSCs without greatly damaging normal cells. We showed that downregulation of Snail by butylidenephthalide resulted in the diminished self-renewal and propagating capacities of oral CSCs (Figure 7). The in vitro and in vivo evidence both support the promising role of butylidenephthalide in oral cancer therapy.

## 4. Materials and Methods

### 4.1. Reagent and Isolation of Cancer Stem Cells

Butylidenephthalide was purchased from Sigma-Aldrich Chemical Co. (St. Louis, MO, USA) and prepared as 50 mg/mL stock solution in dimethyl sulfoxide (DMSO; Merck, Darmstadt, Germany). Butylidenephthalide was further diluted to the final concentrations with the culture medium prior to use.

Oral cancer tissues were isolated from patients recruited in the Oral Medicine Center (Chung Shan Medical University Hospital, Taichung, Taiwan) after obtaining written informed consent, and the characteristics of the oral cancer patients are summarized in Supplementary Table S1. All procedures of tissue acquisition were conducted in accordance with the Declaration of Helsinki and approved by the Institutional Review Committee at Chung Shan Medical University Hospital. Primary oral cancer cell cultures were established as previously described. In brief, after the surgical removal of the oral cancer tissues, the tissues were washed three times in Hanks’ Balanced Salt Solution (HBSS). Samples were obtained and immersed in 0.1% (*w/w*) collagenase- and glucose-containing HBSS for 15 min at 37 °C on a rotating shaker operating at 125 rpm. The collagenase-digested cells were centrifuged at 100× *g* for 10 min and resuspended in a medium, consisting of serum-free Dulbecco’s Modified Eagle Medium (DMEM)/F12 medium (Gibco, Carlsbad, CA, USA) supplemented with N2 supplement (R&D Systems, Minneapolis, MN, USA), 10 ng/mL human recombinant basic fibroblast growth factor (bFGF; R&D Systems), and 10 ng/mL epidermal growth factor (EGF). To identify ALDH1+/CD44+ oral cancer stem cells, we stained cancer cells with the ALDEFLUOR™ assay kit (StemCell Technologies, Vancouver, BC, Canada) and anti-CD44 antibody conjugated to phycoerythrin (Bio-Legend, San Diego, CA, USA) followed by fluorescence-activated cell sorting using a FACSAria II cell sorter (BD Biosciences, San Jose, CA, USA). The sorted ALDH1+/CD44+ cells recovered after fewer than five passages and were tested for mycoplasma contamination. These cells were in the absence for mycoplasma cross-contamination for further experiments. Normal human oral keratinocytes (NHOK) were kindly provided by Dr. Tzong-Ming Shieh (School of Dentistry, China Medical University) and were cultivated in DMEM, as previously described for our study [46,47,48].

### 4.2. Cell Proliferation Assay

NHOK or ALDH1^+^/CD44^+^ oral cancer stem cells were incubated with various concentrations of a butylidenephthalide-containing medium at 37 °C for 48 h followed by MTT ((3-(4,5-dimethylthiazol-2-yl)-2,5-diphenyl tetrazolium bromide) treatment for 3 h. The blue formazan crystals of viable cells were dissolved in DMSO and then evaluated spectrophotometrically at 570 nm. IC_50_ values were calculated by GraFit software (Erithacus Software Ltd., West Sussex, UK) [49].

### 4.3. Flow Cytometry

ALDH1 enzymatic activity was detected using the ALDEFLUOR™ kit according to the manufacturer’s instructions. Cells were stained with a specific ALDH inhibitor, 4-diethylaminobenzaldehyde (DEAB), as a negative control. For CD44 expression, cells were stained with an anti-CD44 antibody conjugated with phycoerythrin. Fluorescence emission from 10,000 cells was assessed with FACSCalibur (Becton Dickinson, Mountain View, CA, USA) using CellQuest software [49].

### 4.4. Transwell Migration and Invasion Assays

Cells were plated into the top chamber of a transwell (Corning, Acton, MA, USA) with a porous, transparent, polyethylene terephthalate membrane (8.0 µm pore size) with lower serum (0.5% [*v/v*] FBS), and a medium supplemented with higher serum was used as a chemoattractant in the lower chamber. After 24 h of incubation, cells on the lower surface of the membrane were stained with crystal violet. The numbers of cells in a total of five randomly selected fields were counted [49].

### 4.5. Colony Formation Assay

A six-well culture dish was coated with an agar–medium mixture (DMEM, 10% (*v/v*) fetal calf serum (FCS), 0.6% (*w/v*) agar). After the bottom layer solidified, the top agar–medium mixture (DMEM, 10% (*v/v*) FCS, 0.3% (*w/v*) agar) containing 2 × 10^4^ cells was added to each well. After 2 weeks of incubation at 37 °C, the number of colonies was counted after staining them with 0.005% crystal violet for five fields per well in triplicate experiments [50].

### 4.6. Bioluminescence Imaging Measurement of Tumor Growth in Nude Mice

All procedures were conducted in accordance with the guidance of the Institutional Animal Care and Use Committee (IACUC) of the Chung Shan Medical University. The 4–5-week-old female immunodeficient nude mice (BALB/c AnN.CgFoxnnu/Crl Narl mice) were obtained from the National Laboratory Animal Center and were used for the xenograft model. The GFP-labeled ALDH1+/CD44+-1 cells were injected subcutaneously into the right front axilla. Then, 10 days post-implantation, the mice were randomly divided into three groups and treated with vehicle alone (Vitamin K, 10 mg/mL), and the experimental group was treated with different doses of BP (100 or 200 mg/kg). An IVIS 50 Imaging Systems (Xenogen Corporation, Alameda, CA, USA) was used for bioluminescence imaging, and the animals were monitored until day 22. The volume was calculated according to the formula, [length × width^2^]/2, and then analyzed using Image-Pro Plus software. Body weight was assessed daily after cell injection [51].

### 4.7. Quantitative Real-Time PCR (qRT-PCR)

Total RNA from cells was isolated from a Trizol reagent according to the manufacturer’s instruction (Invitrogen Life Technologies, Carlsbad, CA, USA). The cDNA was synthesized and amplified using a Superscript III first-strand synthesis system (Invitrogen Life Technologies, Carlsbad, CA, USA). The qRT-PCR reactions were performed on an ABI StepOne TM Real-Time PCR Systems (Applied Biosystems, Foster City, CA, USA). Each target gene was normalized to GAPDH to derive the change in Ct value (∆Ct). The primer sequences used in this study were as follows [52]: Snail: forward primer sequence: 5′-GATGATGGGGTTCTGGCCTC-3′ and reverse primer sequence: 5′-CCCAGATTCACAAAAGCTGCC-3′; Sox2: forward primer sequence: 5′-AACCAGCGCATGGACAGTTA-3′ and reverse primer sequence: 5′-GACTTGACCACCGAACCCAT-3′; GAPDH: forward primer sequence: 5′-AGACCACAGTCCATGCCATC-3′ and reverse primer sequence: 5′-CAGGGCCCTTTTTCTGAGCC-3′.

### 4.8. Western Blot

Proteins’ concentrations in whole cell lysate were quantitated using the Bradford protein assay (Bio-Rad, Santa Rosa, CA, USA) according to the manufacturer’s instructions. Whole cell lysates with 2× Laemmli Sample Buffers (Bio-Rad, CA, USA) were denatured by boiling at 95 °C for 10 min and were subjected to 10% (*w/v*) dodecyl sulfate polyacrylamide gel electrophoresis (SDS-PAGE). Fractionated proteins were then wet-transferred to a polyvinylidene difluoride (PVDF) membrane (Amersham, Arlington Heights, IL, USA) and immersed in the 5% (*w/v*) BSA blocking solution (MilliporeSigma, Darmstadt, Germany). The primary antibodies against Sox2 and Snail were purchased from Santa Cruz Biotechnology, Inc. (Santa Cruz, CA, USA). Following the primary antibodies, the membranes were incubated with the corresponding secondary antibodies. The immunoreactive bands were developed using an ECL-plus chemiluminescence substrate (Perkin-Elmer, Waltham, MA, USA) and captured by LAS-1000 plus Luminescent Image Analyzer (GE Healthcare, Piscataway, NJ, USA) [53].

### 4.9. Overexpression of Snail

Snail was cloned into pLV-EF1a-MCS-IRES-Puro (BioSettia, Cat. No.: cDNA-pLV01; San Diego, CA, USA). Lentivirus production was performed by co-transfection of a plasmid DNA mixture with lentivector plus helper plasmids (VSVG and Gag-Pol) into 293T cells (American Type Culture Collection, Manassas, VA, USA) using Lipofectamine 2000 (LF2000, Invitrogen, Carlsbad, CA, USA) [52].

### 4.10. Self-Renewal Assay

The 10^3^ cells were dissociated and cultured as spheres were plated per well of an ultra-low attachment 96-well plate (Corning, NY, USA) in modified medium consisting of DMEM/F-12 supplemented with N2 (R&D Systems, Minneapolis, MN, USA), 10 ng/mL EGF (Invitrogen, Carlsbad, CA, USA), 10 ng/mL bFGF (Invitrogen), and penicillin/streptomycin. Medium was changed every other day for 2 weeks. The total number of spheroids was counted under a microscope [50].

### 4.11. Statistical Analysis

Three replicates of each experiment were performed. Statistical analysis was carried out by one-way analysis of variance (ANOVA). Tests of differences in the treatments were analyzed by Duncan’s test and a value of *p* < 0.05 was considered statistically significant [50].

## Supplementary Materials

The following supporting information can be downloaded at: https://www.mdpi.com/article/10.3390/ijms23116157/s1.
