# Supplementary material for: Butylidenephthalide Abrogates the Snail-Induced Cancer Stemness in Oral Carcinomas
Source: Int J Mol Sci. 2022 May 31;23(11):6157. doi: 10.3390/ijms23116157 (PMC9180956; doi:10.3390/ijms23116157)
Supplement: Supplementary file 1 [file ijms-23-06157-s001.zip › ijms-1707941-supplementary.pdf]

Supplementary Table S1. Case description and the characterization of oral cancer patients tissue samples

| **Case** | **Age/sex** | **Initial stage** | **ALDH+CD44+ (%)** | **Spheres formation** |
|---|---|---|---|---|
| 1 | 60/M | pT3N2bM0 | 23.4 | Yes |
| 2 | 55/M | pT4N2bM0 | 13.3 | Yes |
